# Dr. Bardsley on Iodine in Bronchocele, Scrofula, and Ascites

**Published:** 1830-01-01

**Authors:** 


### XLIII.

### ON THE INFLUENCE OF IODINE IN BRONCHOCELE, SCROFULA, AND ASCITES. By Dr. BARDSLEY.

In the volume of hospital facts and observations lately published by Dr. Bardsley, of the Manchester Infirmary, some very candid statements are given respecting the powers of iodine. Whatever may be the true explanation of the fact, it is certain that this medicine has not proved of such general and decisive efficacy in ordinary practice, as it seems to have evinced in the hands of those who first introduced it into notice. At the same time, it has shewn such considerable power in arresting the march of disease in many instances, that it would be most desirable to ascertain in what cases it is likely to prove of service, as well as those in which it is not. Let us glance at its effects in bronchocele.

Dr. Bardsley gives a table, which shews that he has employed the iodine in thirty cases of bronchocele. Of the thirty, nine, or nearly one in three, were cured, and in none of these had the disease existed for more than two years. Six received some benefit, and the remaining fifteen were not at all relieved.

"In several of the above instances (which are selected from some others)

it must be allowed that the iodine failed to produce any diminution of the tumours, though its exhibition was regularly persevered in for many months, and the dose of the medicine gradually increased to as great an extent as the state of the stomach and strength of the patients would allow. My experience of the powers of iodine is opposed to the following statement of Dr. Gairdner: 'It seldom fails of effecting a complete cure, and when it does, it almost always reduces the swelling very considerably.'* In some cases large tumours have been much diminished in a short space of time under the external and internal use of iodine, but *in not a few instances* the beneficial influence of this remedy has been solicited in vain. Iodine will unquestionably be found a valuable medicine in some examples of bronchocele, but it is by no means entitled to the character of a specific in that affection."

We fancy that these opinions will accord pretty nearly with the experience of most unbiassed practitioners. Dr. Bardsley is led to think, from the results of his trials, that iodine is a remedy at least of equal, if not superior, efficacy, to any of the numerous substances that have been proposed for the cure of scrofula. He has seen it succeed, on several occasions, in removing enlarged scrofulous glands, after the failure of other plans of treatment. Dr. Bardsley has also been anxious to test its *real virtues* in a tuberculous state of the lungs. Need we mention the results of such experiments? Our author has derived little or no benefit from iodine in paralysis; nor, except in two cases, has he ever witnessed any good effects from it in chorea.

"I have also made trial of iodine in chorea, but never witnessed any good effects from it, except in two cases. I am at a loss to account for the difference between the results of my experiments with iodine in paralysis and chorea, and those of Dr. Manson, for in his hands this remedy has proved almost uniformly successful. I wish, however, explicitly to remark, that I place the greatest reliance on the accuracy of Dr. Manson's observations, and his known candour and respectability of character entitle his statements to confidence. It is worthy of remark, that with several young females labouring under chorea, to whom I have administered the iodine for some time, the menses have not made their appearance until the sixteenth year. This I merely throw out as a conjecture, whether the action of the medicine in question upon the uterus could have any effect in retarding menstruation. It is a well-established fact, that iodine exerts a powerful action on the glandular system, for the mammæ occasionally undergo considerable diminution in size during its use. This is a point of some importance in the selection of this remedy for females."

In ascites depending on *supposed* enlargement of the liver iodine has appeared to our author to be a medicine of great efficacy, and the following cases are adduced in support of his opinion.

*Case* 1. Edward Placey, ætatis 40, applied at the Infirmary about the end of August, 1826, with a considerable quantity of fluid in the abdomen—occasional pain in the right hypochondrium—loss of appetite and strength—sallow countenance—urgent thirst—and feeble pulse. He had formerly indulged to excess in the use of spirits, and laboured under the foregoing symptoms for about eight months. After the removal of the pain in the hypochondrium by the use of a few leeches and a blister, ten drops of the solution of the hydriodate of potass (thirty grains of the salt to an ounce of distilled water) were ordered to be taken thrice daily. When the blistered surface had healed half a drachm of an ointment composed of two scruples of the hydriodate to an ounce of axunge, was rubbed in night and morning. In the course of six weeks an evident amendment occurred, the urine became more copious, the thirst less troublesome, and the patient's strength improved. The dose of the solution was gradually increased to twenty drops three times a day, and friction with the ointment

* "Vid. opus ante cit., p. 35."

was at the same time continued. At the end of three months Placey was discharged cured, without having experienced any unpleasant effects from the use of the iodine.

"*Case* 2. James Pilkington, 35 years of age, came under my care in June, 1827.

"He had enjoyed a tolerably good state of health until December, 1826, when he noticed a slight fulness of the abdomen. On his first appearance at the Infirmary, the liver was found to be somewhat enlarged, but not at all painful on pressure, and there was a very perceptible fluctuation of water in the belly. His countenance was pale, and appetite and strength much impaired. As I was desirous of giving the iodine a fair trial, I commenced at once with that medicine, in the same proportion as was employed in the preceding case. This plan was pursued with little variation until the 16th of August, without any marked benefit. Twenty drops were now ordered to be taken three times a day, and a drachm of the ointment to be used morning and evening. The report of the 24th of September, as taken verbatim from my register, states, 'that an increased flow of urine, and considerable diminution in the dropsical swelling, had been produced by this medicine.' The iodine was exhibited until the 4th of November, when the patient wished to be discharged, in order to resume his usual occupation as a carter."

*Case* 3. William Arnold, æt. 50, became an out-patient on the 24th of March, 1828, with fluctuation in the belly; the hardened edge of the right lobe of the liver extending into the epigastrium; complexion slightly yellow; spirits much depressed; pulse feeble; bowels costive; urine scanty and high-coloured. He had laboured under loss of appetite and debility for more than six months, but had only observed the swelling in the belly for two. The same prescription as in the foregoing case was employed, and the iodine continued with only slight benefit till the middle of June, when an obvious improvement occurred, and continued to increase till the end of August, when the dropsical swellings had entirely disappeared, and recovery was complete. He was discharged cured, and has since enjoyed good health.

"*Case* 4. Joseph Major, 43 years of age, applied to me on the 4th of July, 1828, on account of an ascites which had existed for nearly six months.

"He had a slight cough, with little expectoration, and some difficulty of breathing. The urine was diminished in quantity, and high coloured. The pulse was feeble and strength impaired. The right lobe of the liver was somewhat enlarged and indurated. The alvine evacuations were tolerably natural. He had taken a variety of diuretics and mercury, but with very little effect. A blister was applied to the chest, and an ordinary linctus ordered for the cough. August 1st. The respiration has become free, and the cough has entirely disappeared. He was now directed to take the iodine in the manner before noticed. September 7th. No amendment has as yet resulted from the medicine. Ordered to continue. October 22d. During the last week, he has passed much more water and felt rather stronger. To persevere with the iodine. November 24th. The swelling in the belly is considerably reduced; and so much benefit has already accrued from the remedy as to encourage a further trial of it. December 4th. He has persisted in the use of the iodine since the last report, and without perceiving any ill effects from it. The dropsical effusion is quite gone, and his health is better than it has been for the last few years."

*Case* 5. Sarah Fielding, æt. 49, of intemperate habits, applied in June 1828, with evident ascites and an unnatural fulness about the region of the liver, which, however, was not tender upon pressure. Her countenance was sallow and her bowels costive. She dated her complaints to a severe attack of fever in August 1827, since which she had never recovered her health; the enlargement of the abdomen with scantiness of urine had existed for about two months, and followed an attack of pain in the right side and loins. After obtaining a regular action of the intestinal canal by the aid of mild purgatives, iodine was employed as before. On the 2d of August her urine was sensibly increased, and the collection of fluid in the belly was somewhat diminished. This slight amendment encouraged her to persevere in the use of the medicine; by the 4th of November she felt quite well; and so she still remains.

The remarks of Dr. Bardsley on these cases are so sensible and so candid that we have nothing further to say, than that we perfectly agree with him.

"The preceding examples, it must be admitted, are too few to establish the powers of iodine in dropsy, but they serve to shew that occasional advantage may be derived from that remedy, in some instances of dropsical effusion, where there is reason to suspect obstruction to the free return of the blood. It is very possible that the benefit in these cases may have arisen in a different manner from what I have supposed, or that it might have been produced by other means; but still that great good was derived from the iodine, cannot for a moment be doubted. In dropsies proceeding from disease of the lungs, the heart, or its large vessels, scirrhous enlargement of the liver and spleen, and some other causes, our art can afford but little service; still it is more particularly in ascites depending upon some enlargement of the liver, or the presence of steatomatous tumors in the abdomen, that iodine is likely to afford some chance of relief. Dr. Abercrombie says, that he has seen very good effects from the external use of iodine in several cases of chronic affections of the liver.* I have also used this medicine in cancerous affections of the mammæ and uterus, but with an unsuccessful result. In the whole of the above trials with iodine, I have employed internally a solution of hydriodate of potass in the proportion of half a drachm to an ounce of distilled water; and as an external application, two scruples of the salt to an ounce of axunge. The dose of the former has been ten drops twice and thrice a day, gradually increased to twenty, at the same intervals; and of the latter, a drachm has been directed to be rubbed in over the part affected night and morning. It is scarcely necessary to add, that caution must be observed in the internal exhibition of iodine, and the dose must be gradually increased; for, like other powerful medicines, if heedlessly employed, it is apt to occasion serious consequences."

* "Pathological and Practical Researches on Diseases of the Stomach, the Intestinal Canal, the Liver, &c. p. 360."

